# Lipectomy as an alternative for superficialization of autologous AVF in obese patients: experience of a referral center in Amazon

**DOI:** 10.1590/1677-5449.202300542

**Published:** 2024-03-25

**Authors:** José Maciel Caldas dos Reis, Flávio Roberto Cavalleiro de Macêdo Ribeiro, Glauco dos Santos Melo, Humberto Balbi Reale, Mariseth Carvalho de Andrade

**Affiliations:** 1 Fundação Hospital de Clínicas Gaspar Vianna - FHCGV, Serviço de Cirurgia Vascular, Belém, PA, Brasil.; 2 Centro Universitário Metropolitano da Amazônia - UNIFAMAZ, Belém, PA, Brasil.; 3 Universidade do Estado do Pará - UEPA, Belém, PA, Brasil.

**Keywords:** lipectomy, arteriovenous fistula, obesity, vascular access, superficialization, hemodialysis

## Abstract

**Background:**

The preferred vascular access for hemodialysis is a native arteriovenous fistula (AVF) because it offers the best results in the short and long terms, lower morbidity and mortality, and has additional advantages in relation to central venous catheters or arteriovenous grafts. However, obesity can present an additional challenge because of the barrier of subcutaneous cellular tissue covering the surface of the vein to be punctured.

**Objectives:**

The authors review their experience with excision of subcutaneous tissue (lipectomy) overlying upper arm cephalic vein arteriovenous fistulas in obese patients.

**Methods:**

Consecutive vascular access patients undergoing lipectomy for cannulation with difficult access because of vein depth were reviewed. Cephalic vein depth was measured by ultrasound in all cases.

**Results:**

Twenty-two patients were reviewed (15 men and 7 women), with a mean body mass index of 34.0 kg/m^2^ (range: 28-40 kg/m^2^). Mean age was 58.4 years. The mean preoperative vein depth of 7.9 mm (range: 7.0-10.0 mm) was reduced to 4.7 mm (range: 3.0-6.0 mm) (*P* 0.01). The mean follow-up period for patients was 13.2 months. Four patients were lost to follow-up and four died during the period due to causes unrelated to vascular access.

**Conclusions:**

Obesity should not be a limiting factor to creation of a native AVF, since lipectomy is a relatively simple option for superficialization, enabling functioning native and deep arteriovenous fistulas in obese patients.

## INTRODUCTION

The preferred vascular access for hemodialysis is a native arteriovenous fistula (AVF) because it offers the best results in the short and long terms, lower morbidity and mortality, and has additional advantages in relation to central venous catheters or arteriovenous grafts.^1-4^

The growing prevalence of obesity is a well-known problem worldwide, as is the relationship between obesity and end-stage kidney disease (ESKD).^1,5^ Obesity is clearly linked to type 2 diabetes, hypertension, and dyslipidemia, which are also known and growing causes of kidney failure.^1-5^ While the best treatment for ESKD is kidney transplantation, hemodialysis, which requires vascular access, is a key element in management of ESKD.^1^ However, obese patients have problems with access maturation associated with deep or tortuous upper limb veins, which can lead to significant and inconvenient complications.^1,4,6^

The National Kidney Foundation guidelines recommend following the “rule of 6”, according to which the fistula should be mature 6 weeks after creation, should have flow greater than 600 mL/min, and should be at least 6 mm in diameter and the distance between the vein and the surface of the skin should be 6 mm. It is the last of these criteria that is impacted negatively by obesity.^7,8^

**Figure 1:**
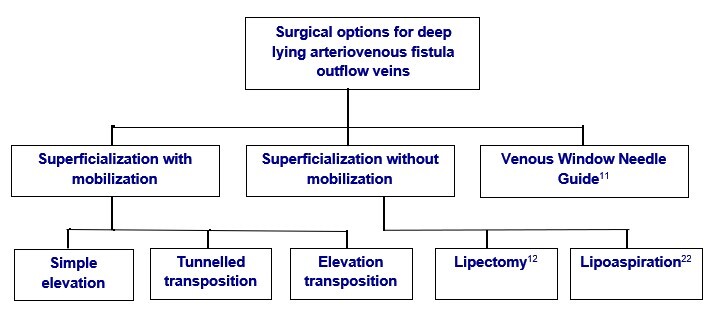
Surgical options to enable cannulation of deep arteriovenous fistula run-off veins in upper limbs.

While current guidelines do not define a body mass index (BMI) cutoff for contraindication of fistula creation, obese patients are less likely to have an autogenous access and tend to need some type of intervention to facilitate cannulation of the access.^7^ A number of options have been described in the literature: superficialization with tunnel transposition, simple elevation of the vein, or lipectomy by excision of fatty tissue or liposuction according to a method adapted by Cs Nagy et al.^8^ (Figure 1). Placement of a subcutaneous guide (Venous Window Needle Guide^®^ [VWING]; Vital Access Corp, Salt Lake City, Utah) has also been reported.^8-11^

This study employs the lipectomy concept as described by Bourquelot et al.,^12^ which is a currently acceptable method of superficialization of the cephalic vein for native AVFs. It constitutes a relatively simple and low-cost procedure to achieve reliable access cannulation in obese patients.^12^

The objective of this study is to review a single service’s experience with lipectomy for autologous accesses involving the cephalic vein in the forearms and arms of obese patients.

## METHODOLOGY

### Study design

This is a cross-sectional, retrospective, and descriptive study conducted at the vascular surgery service at the Hospital de Clínicas Gaspar Vianna (HCGV). The research corpus was extracted from a database of consecutive AVF creation procedures conducted for hemodialysis at a public tertiary hospital in the city of Belém, Pará state, Brazil. The aim of the study is to describe the profile of the patients and the safety and efficacy of lipectomy as an option for AVF superficialization in obese patients at a referral center in The Amazon region. The methodology employed complies with the principles set out in “Guidelines on good publication practice” from the Committee on Publication Ethics (COPE).^7^

### Sample size calculation

The sample size was defined using the formula for finite populations,^13,14^ with test power of 0.95, considering an event probability of 0.5 (or 50%), and adopting a significance level of α = 0.05. During the period considered as the parameter (60 months), a total of 328 patients were seen and so for the 3 months data collection period, the number of patients expected would be 18. The formula indicated a minimum sample size of 17 patients.

### Ethical considerations

The project was submitted in advance to the Research Ethics Committee (CEP - HCGV) and complies with National Health Council (Conselho Nacional de Saúde) Resolution 466/2012, protecting confidentiality and anonymity. The study was approved by the CEP with opinion number 4.263.413 and Ethics Appraisal Submission Certificate: 36268220.8.0000.0016.

### Study population

**Figure 2:**
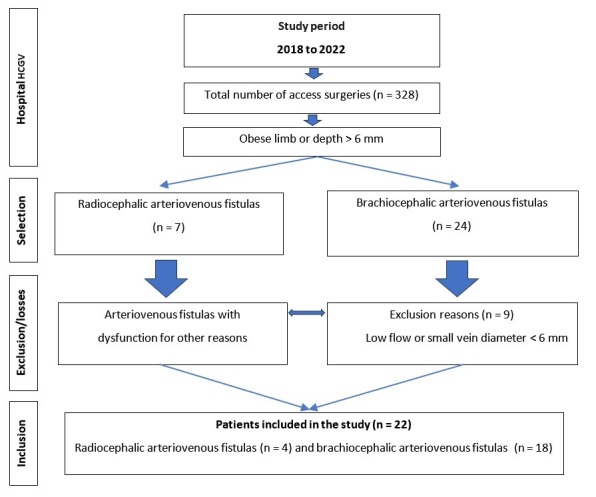
Flow diagram illustrating selection, exclusion, and inclusion of cases.

We recruited all chronic dialytic renal patients registered at the institution who agreed to participate (and signed a free and informed consent form) with dysfunctional radiocephalic or brachiocephalic native AVFs because of limb obesity or vein depth greater than 6 mm. We excluded patients with AVFs that were dysfunctional for other reasons, as illustrated in the STROBE flow diagram in Figure 2. This study was limited to radiocephalic and brachiocephalic fistulas constructed from January 2018 to December 2022.

### Assessment of patients

In line with the routine practice at the service, initial assessment consisted of taking patient history and a physical examination including inspection of the upper extremities for edema and pulse asymmetry, arterial blood pressure measurement, and the Allen test. All cases routinely have a Doppler ultrasound examination before creation of the access.

### Surgical procedures

**Figure 3:**
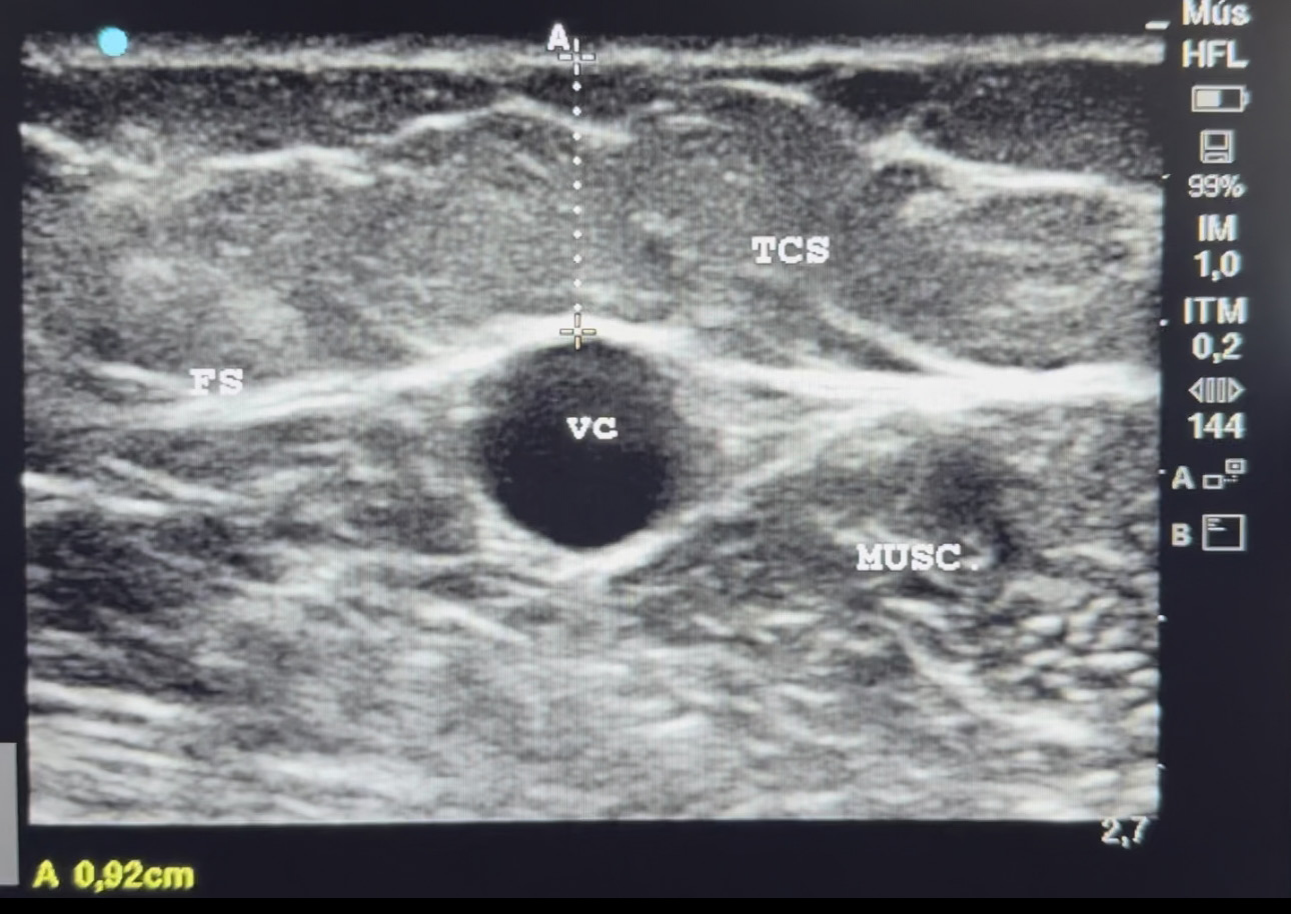
Preoperative ultrasonographic appearance: transverse image including section from skin to the cephalic vein in the arm. The broken line indicates the skin-vein distance exceeding 6 mm. The letters mark examination findings: FS: superficial fascia; TCS: subcutaneous cellular tissues; VC: cephalic vein; MUSC: muscle.

In all patients, the access procedure was performed in two stages. First an autologous AVF is created in the forearm (radiocephalic) or cubital fossa (brachiocephalic). In cases with early access failure (due to thrombosis), a second fistula is created a few centimeters proximal to the first, unless advanced atherosclerotic lesions or small arterial diameter are encountered. In these cases, we use the brachial artery at the cubital fossa. Before the second stage, patients undergo ultrasound assessment to acquire information about the diameter and depth of the vessel (Figure 3).

**Figure 4:**
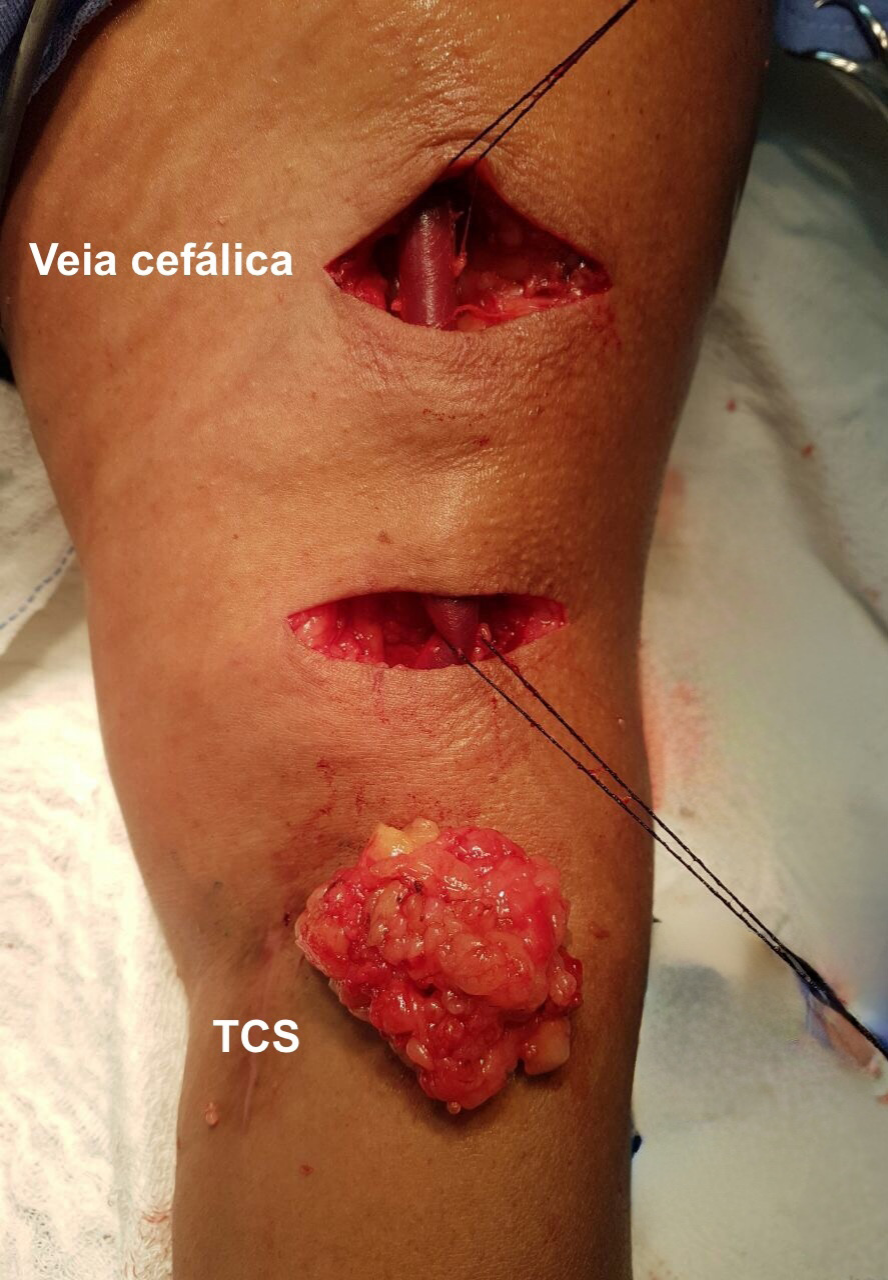
Intraoperative image showing incisions for lipectomy over a brachiocephalic arteriovenous fistula in the left upper limb and the recently removed fat pad.

Stage two is performed 4 to 8 weeks after formation of the fistula and consists of a lipectomy procedure to achieve superficialization of the vein and enable safe puncture. The procedure was performed with two transverse incisions, approximately 5 cm each, as described by Bourquelot et al.,^12^ and removal of the fat pad (Figure 4). Preventative hemostasis with Esmarch bandage or pneumatic torniquet was not employed. For the second stage, all patients were administered an anesthetic protocol with plexus block and sedation and a prophylactic dose of antibiotics. Finally, we routinely employ a 3.2 mm continuous closed suction drain (Portovac^®^) for 24 to 48 hours and closure is with non-absorbable separated sutures, which completes the procedure.

### Patient follow-up

**Figure 5:**
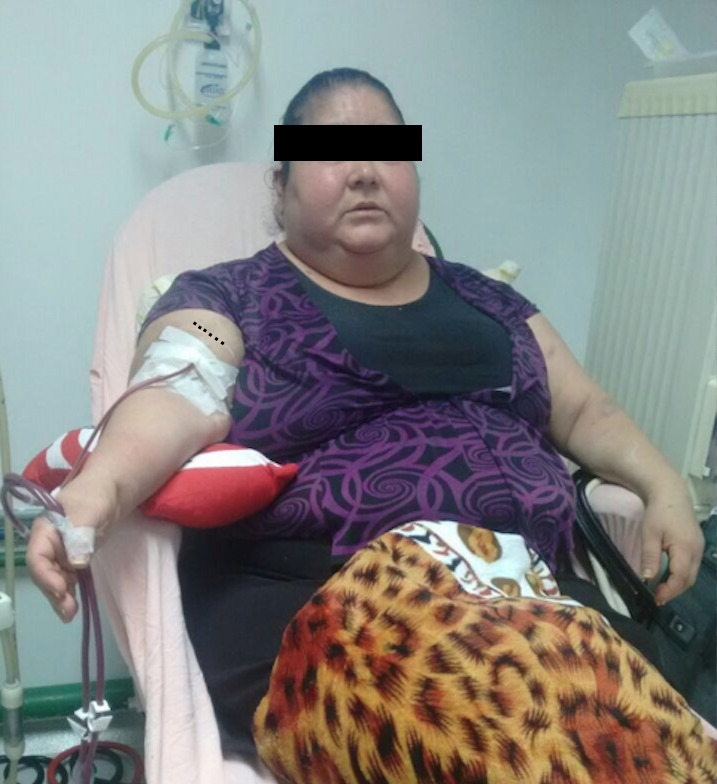
Patient with grade I obesity on 28th postoperative day after lipectomy, already using the access. The dots indicate the already healed lipectomy incision.

Routine follow-up examination for all patients with an AVF who have undergone lipectomy includes a brief ultrasound assessment of vein diameter, location, and flow volume, before the access is cleared as ready for use. Vein depth measurements were repeated at the same locations for each patient after the intervention and the new mature access was mapped and marked for the nursing team responsible for cannulation. Figure 5 shows a patient on the 28th postoperative after lipectomy, already using the access.

### Data collection

The following data were collected: sex, age, hemodialysis duration, number of previous accesses, type of fistula, BMI, time until access was cleared for use, mean vein diameter and depth on ultrasound, complications, and patency.

### Statistical analysis

Information on sample characteristics was collected and organized in a Microsoft Excel^®^ spreadsheet. Statistical analysis was conducted to assess the results of the variables in the sample using the G test and the chi-square test of adherence for univariate tables. Descriptive and analytical statistics were computed using BioEstat^®^ version 5.4. For decision making, a significance level of α = 0.05 (or 5%) was adopted, marking significant values with asterisks (*).

## RESULTS

This retrospective study recruited 22 obese patients (15 men and 7 women), selected from a group of 328 patients who underwent creation of an autogenous AVF from January 2018 to December 2022. In all cases, creation of the AVF was considered successful intraoperatively.

The artery supplying functioning AVFs was the proximal radial artery in 4 patients (18.2%) and the brachial artery in 18 (81.8%). None of the patients had steal syndrome requiring intervention. Male patients predominated and mean age was 58.4 years. The most prevalent cause of end-stage kidney disease was diabetes mellitus in 59%, followed by hypertension in 38% of the patients. Comorbidities were present in 95.5%, the most common of which were hypertension (63.6%), diabetes (54.5%), and heart disease (31.8%). Mean time on hemodialysis was 4 months and 88.8% of the patients had the superficialization procedure performed on their first autogenous access. Mean BMI was 34 kg/m^2^, and the majority had a grade I obesity nutritional classification. Mean time between creation of the access and lipectomy was 45.1 days, and the time from intervention (lipectomy) to use of the access for hemodialysis oscillated from 21 to 42 days, with a mean of 30.9 days. Six patients (22.7%) had had one unsuccessful attempt to create a distal access prior to the reference procedure. In 14 patients (63.6%), lipectomy was performed before any attempts at puncture, whereas 8 patients (36.3%) underwent unsuccessful cannulation attempts before being referred for the procedure (stage two).

Complications, all with low morbidity, were observed in nine patients (40.9%), the most common of which were seroma (22.7%) and hematoma (22.7%). Two patients had superficial skin necrosis and one had a hemorrhage and required surgery. There were no cases of wound infection or dehiscence. Three patients had their first cannulation delayed because of hematoma and one because of significant seroma. On average, lipectomy was performed 45.1 days after access creation (range: 27.0 - 90.0). Mean preoperative cephalic vein depth within the limb was 7.9 mm (range 7.0 - 10.0 mm). This was reduced to a mean depth of 4.7 mm (range: 3.0 - 6.0 mm).

Mean patient follow-up was 13.2 months. There were four deaths during follow-up, from causes unrelated to vascular access (two from acute myocardial infarction, one after ischemic stroke, and one from infectious complications in the intensive care unit), and four patients were lost to follow-up. Three patients had endovascular interventions to maintain dialysis access. At 12 months, primary patency after lipectomy was 66.6%, and secondary patency was 75%. The patients’ profile is detailed in Tables 1 and 2 along with data related to the accesses analyzed for the study.

**Table 1:** Profile of patients who underwent lipectomy from January 2018 to December 2022 at the Hospital de Clínicas Gaspar Vianna (HCGV), Belém, Pará, Brazil.

**Variables**	**Frequency**	**% (N = 22)**
Sex		
Female	7	31.8%
Male*	15	68.2%
Age group		
< 50	4	18.1%
50 to 59	8	36.4%
60 to 69	10	45.5%
Minimum/mean/maximum	33/58.4/69	
Nutritional classification		
Overweight	2	9.1%
Grade I obesity**	13	59.1%
Grade II obesity	6	27.3%
Grade IIII obesity	1	4.5%

* p = 0.0004, chi-square test of adherence;

** p = 0.0015, G goodness of fit test.

**Table 2:** Description of variables related to types of access, pre-intervention and post-intervention depths until access cleared for use, and follow-up of lipectomy cases from January 2018 to December 2022 at the Hospital de Clínicas Gaspar Vianna (HCGV), Belém, Pará, Brazil.

**Variables**	**Frequency**	**% (N = 22)**	**p**
Types of arteriovenous fistula (AVF)			0.0028*
Brachiocephalic*	18	81.8%	
Radiocephalic	4	18.2%	
Pre depth (mm)			
Min./mean/max.	7.0/7.9/10.0		< 0.0001**
Post depth (mm)			
Min./mean/max.	3.0/4.7/6.0		
Time from AVF to lipectomy (days)			------
Min./mean/max.	27.0/45.1/90.0		
Time from lipectomy to puncture (days)			------
Min./mean/max.	21.0/30.9/42.0		
Follow-up (months)			------
Min./mean/max.	2.0/13.2/22.0		

* G goodness of fit test;

** Paired Student’s *t* test.

## DISCUSSION

The World Health Organization estimates that 2.3 billion people will be overweight and 700 million will be obese by 2025.^15^ A recent report suggests that if the rate of increase in overweight and obesity continues at the present pace, approximately 85% of the United States population will be overweight or obese by 2030, reaching 100% by 2048.^16^

These data are alarming, because the close relationship between obesity, arterial hypertension, and diabetes is intimately related to development of renal diseases, including chronic kidney disease (CKD).^5,14,17-19^ In Brazil, men and women are equally affected and obesity is already close to epidemic proportions, with approximately 20% of adults with BMI greater than or equal to 30 kg/m^2^. There are no official data for the Amazon region on the prevalence of end-stage CKD vs. obesity, but the mean rates of overweight and obesity are similar to the mean rates in the other states of Brazil.^5^

Obese patients and diabetic patients are part of a group at increased risk of autologous AVF placement.^1-4,18,19^ A mature AVF that is functioning well, but cannot be cannulated because of limb obesity, does not provide any benefit and contributes to prolonged catheter dependence and its potential complications, causing frustration for the patient and the health care team.^6-12,18,19^ This study observed a mean preoperative cephalic vein depth of 7.9 mm, which was reduced to a mean depth of 4.7 mm at post-intervention follow-up. Complications occurred in nine patients (40.9%), all considered of low morbidity and none of which caused delays in the access being passed as ready for use.

Obese patients often need additional procedures because of vein depth, in order to enable adequate AVF maturation.^2-4,19^ This is compounded by higher failure rates because of thrombosis or dysfunction.^4,18^ Paradoxically, for the same reasons, many obese patients have cephalic veins considered adequate for a fistula that have been spared repeated injury before dialysis because of their inaccessible location.^4,11,19,20^ Ultrasound enables precise, safe, and convenient assessment of whether these veins can be used for creation of autogenous accesses.^19-21^

The National Kidney Disease Outcomes Quality Initiative and Fistula First guidelines endorse the “rule of 6s”, by which an AVF should have a diameter greater than or equal to 6 mm, a depth less than 6 mm, and fistula flow exceeding 600 mL/min. Additionally, a 6cm functional cannulation zone length is also often recommended.^7,11^ Therefore, the recommendation for reliable access cannulation is a maximum vein depth of 6 mm.^11^

Superficialization of the arterialized vein to facilitate cannulation of the AVF can be achieved with several techniques, such as tunnel transposition, simple elevation of the vein, or lipectomy.^1,9,11-19,22^ Placement of a subcutaneous needle guide (VWING) was also reported recently by Hill et al.^23^

The concept of minimally invasive lipectomy was introduced by Zeindler et al.^24^ They presented endoscopic liposuction as a technical option for reducing the adipose layer over the vein to be punctured, even though this is a case series, this group of authors report results that attract attention, since they describe a scenario of access dysfunction in 12 obese patients with a mean venous depth of 10.1 mm.

Although the technique was initially associated with wound necrosis and large hematomas, nowadays it is seen as an option for correction of access dysfunction in obese patients. It is intended to minimize tissue trauma and make good functional and esthetic results more likely.^25^ Moreover, it also offers advantages over conventional superficialization methods: fewer incisions and the potential to reduce wound complications. However, certain issues related to the procedure must also be considered, including the cost, the need for endoscopic surgery skills, and the associated learning curve, since it is not part of the arsenal of many vascular surgeons in Brazil.^4^

There is a rising trend towards minimally invasive interventions in many specialties. In the case of vascular surgery, the tendency is to adopt endovascular procedures, although there is also potential for synergy between techniques. Therefore, it is speculated that in the future percutaneous AVFs with endoscopic superficialization could become an option for obese patients with ESKD.^1^

With the technique proposed by Bourquelot et al.^12^ in 2009, the cephalic vein is left intact in its native position, reducing the risk of twisting or kinking a repositioned vein. Excellent primary and secondary 1-year patency results have been reported, at 71 and 98%, respectively.^12^ Finally, the use of locoregional anesthesia in the majority of obese patients with end-stage CKD is a major advantage. This was the method of choice adopted in 100% of the procedures conducted at this service.

While the current European Society for Vascular Surgery (ESVS) guidelines do not define a specific BMI level that is a contraindication for fistula creation, the ESVS recommends a primary or secondary surgical procedure to facilitate maturation, because the long-term patency of AVFs in the obese population is worse than in the non-obese population and secondary failure rates are higher.^1,4,7^

Tordoir et al.^6^ conducted a systematic review of the literature on different techniques to facilitate cannulation of autogenous hemodialysis AVFs. They selected 17 studies on treatment of cannulation complications and found that lipectomy had an initial success rate of 100% with primary and secondary patency of 71 and 98%, respectively, at 1 year follow-up.^6^ In the present study, primary patency was defined as the length of time (in months) with uninterrupted patency with no need for intervention.^20-22^ Primary assisted patency was the duration of uninterrupted patency after initial construction of the AVF in cases in which some type of additional interventional procedure was necessary. In turn, cumulative patency (secondary) was the length of time from original construction of the AVF, during which AVF use was interrupted because of thrombosis, with or without recovery of the AVF, until the access was abandoned.^12,20-22^ The primary, primary assisted, and secondary patency rates during the study period were 66.6, 70.2, and 75% respectively.

The obesity rate in the hemodialysis population is approximately double the rate in the general population and lipectomy is a technical resource adopted to improve cannulation success in this group of patients.^6,9-12,17,20,21^ However, some patients in the overweight range, with BMI around 28 kg/m^2^, will also undergo the procedure because of the excessive distance between the skin and the cephalic vein. In our sample, this occurred in 9.1% of cases and in proximal accesses at the cubital fossa, where the cephalic vein runs relatively deep as it approached the elbow or when the expected dilation of the vein appears to be being prevented by a thick superficial fascia trapping the vein.

There is no consensus in the literature on primary lipectomy or versus as a second stage. Elbarbary^10^ studied the viability and safety of lipectomy performed during the same session as creation of the hemodialysis fistula in patients with deep cephalic veins, compared with secondary lipectomy. Immediate technical success was achieved in all cases in both groups. Clinical success was not significantly different between the groups.^10^ The cumulative primary patency rate was 88% at 1 year, in line with second-stage lipectomy results. The majority of postoperative complications were mild, with no significant difference between primary and secondary lipectomy.^21-24^

The majority of authors report improved diameter and depth and good patency after secondary superficialization procedures, but Elbarbary^10^ have questioned the time elapsed from creation of the fistula until successful cannulation, which can result in longer time using a central venous catheter. This is especially important in countries where patients are referred for surgery immediately before or just after starting hemodialysis, which is not the case in the Amazon region, where the cases operated had been on hemodialysis for an average of 4 months. In the present study, in which 100% of lipectomies were second-stage, the mean time using a catheter was 76 days.

Primary lipectomy has the additional advantage of operating in a virgin area, avoiding dissection over an adherent vein with subjacent hematoma or fibrosis from previous attempts at cannulation, as observed in some cases of secondary lipectomy.^9^ This occurred in eight cases in the present series (36.3%) and can cause postoperative complications because of difficulties during dissection.

For these reasons, indication of lipectomy is not consensus in the literature, but it does appear to be preferred as a second-stage intervention, only performed if clinical and imaging follow-up shows that superficialization is needed. Moreover, superficialization of an AVF appears to be easier to execute when the diameter of the vein has already increased and the vein wall has thickened as part of the arterialization process.

The results of the present article should be interpreted in the context of its limitations. The study is retrospective, with a relatively short follow-up period, no comparison group, and a risk of selection bias. For this reason, it is not possible to make a general recommendation that all obese patients with deep AVFs should undergo lipectomy.

This study presents the experience of a referral service in the Amazon region and summarizes the available evidence on superficialization methods with lipectomy in studies that deal with obese patients. It also offers specialists working in Brazil the opportunity to widen the range of procedures available for accesses that are dysfunctional because of limb obesity and emphasizes the importance for surgeons considering these aspects to plan and mange patient expectations.

## CONCLUSIONS

Obesity should not be a factor limiting creation of a native AVF, since lipectomy is a promising option, that is relatively simple and constitutes an important element in the surgical superficialization arsenal, enabling functionality of deep native AVFs in this population. In the experience of this service, the mean time between creation of the access and the lipectomy procedure was 45 days, plus a further 30 days until the access was cleared as ready for cannulation. The procedure achieved the primary objective of reducing the thickness of the fat pad between the skin and the vein from an average of 7.9 mm to a mean of 4.7 mm (less than 6 mm).
