# Supplementary material for: Lipectomy as an alternative for superficialization of autologous AVF in obese patients: experience of a referral center in Amazon
Source: J Vasc Bras. 2024 Mar 25;23:e20230054. doi: 10.1590/1677-5449.202300542 (PMC10984606; doi:10.1590/1677-5449.202300542)
Supplement: Translation

# Translation: Lipectomia como alternativa de superficialização de fístula arteriovenosa autóloga em pacientes obesos: a experiência de um centro de referência na Amazônia

**DOI:** 10.1590/1677-5449.202300541

**Authors:** José Maciel Caldas dos Reis, Flávio Roberto Cavalleiro de Macêdo Ribeiro, Glauco dos Santos Melo, Humberto Balbi Reale, Mariseth Carvalho de Andrade

**Affiliations:** 1 Fundação Hospital de Clínicas Gaspar Vianna - FHCGV, Serviço de Cirurgia Vascular, Belém, PA, Brasil.; 2 Centro Universitário Metropolitano da Amazônia - UNIFAMAZ, Belém, PA, Brasil.; 3 Universidade do Estado do Pará - UEPA, Belém, PA, Brasil.

**Keywords:** lipectomia, fístula arteriovenosa, obesidade, acesso vascular, superficialização, hemodiálise

## Abstract

**Contexto:**

O acesso vascular preferencial para hemodiálise é a fístula arteriovenosa nativa, pois oferece melhores resultados em curto e longo prazo, proporciona menor morbimortalidade e traz vantagens adicionais em relação aos cateteres venosos centrais ou enxertos arteriovenosos. No entanto, a obesidade pode oferecer um desafio adicional proporcionado pela barreira de tecido celular subcutâneo que recobre a superfície da veia a ser puncionada.

**Objetivos:**

Demonstrar a experiência do serviço com a lipectomia em acessos autólogos em pacientes obesos.

**Métodos:**

Foram revisados ​​pacientes consecutivos submetidos à lipectomia por impossibilidade ou dificuldade na punção da FAV, motivada pela acentuada profundidade da veia cefálica no antebraço ou braço.

**Resultados:**

Vinte e dois pacientes foram revisados (15 homens e 7 mulheres), com índice de massa corporal médio de 34 kg/m^2^ (variação de 28 a 40 kg/m^2^). A idade média foi de 58,4 anos. O tempo médio entre a confecção do acesso e a lipectomia foi de 45,1 dias, e o tempo da intervenção até a liberação para uso nas sessões de hemodiálise oscilou de 21 a 42 dias, com média de 30,9 dias. A profundidade média pré-operatória da veia cefálica no membro foi de 7,9 mm (variação de 7,0 a 10,0 mm). Isso foi reduzido para uma profundidade média de 4,7 mm (faixa de 3,0 a 6,0 mm) (P = 0,01). O período médio de seguimento dos pacientes foi de 13,2 meses. Houve perda de seguimento em quatro pacientes e quatro óbitos no período não relacionados ao acesso vascular.

**Conclusões:**

A obesidade não deve ser um fator limitante para a criação de uma FAV nativa, pois a lipectomia é uma alternativa relativamente simples de superficialização, que permite a funcionalidade de fístulas arteriovenosas nativas e profundas em obesos.

## INTRODUÇÃO

O acesso vascular preferencial para hemodiálise é a fístula arteriovenosa (FAV) nativa, pois oferece melhores resultados em curto e longo prazo, proporciona menor morbimortalidade e traz vantagens adicionais em relação aos cateteres venosos centrais ou enxertos arteriovenosos^1-4^ .

A crescente prevalência de obesidade é um problema global bem conhecido, assim como a relação entre obesidade e doença renal em estágio terminal (DRT)^1,5^. A obesidade está claramente relacionada a diabetes tipo 2, hipertensão e dislipidemia, que são também causas conhecidas e crescentes de falência renal^1-5^. Embora o melhor tratamento para DRT seja o transplante renal, a hemodiálise, que depende do acesso vascular, é um elemento-chave no manejo da DRT^1^. No entanto, os pacientes obesos apresentam problemas de maturação associados a veias profundas ou tortuosas nos membros superiores, podendo levar a complicações significativas e inconvenientes^1,4,6^ .

As diretrizes da National Kidney Foundation sugerem, conforme a “regra dos 6”, que a fístula está maturada após 6 semanas de confecção e deve ter fluxo maior que 600 mL/min e pelo menos 6 mm de diâmetro e 6 mm de distância entre a veia e a superfície da pele. É exatamente no último quesito que a obesidade impacta negativamente^7,8^ .

Embora as diretrizes atuais não definam estritamente um nível de índice de massa corporal (IMC) que seja uma contraindicação à criação de fístulas, os pacientes obesos são menos propensos a ter um acesso autógeno confeccionado e acabam requerendo algum tipo de intervenção para facilitar a canulação do acesso^7^. Algumas opções encontram-se disponíveis na literatura: superficialização com transposição de túnel, elevação simples da veia ou lipectomia por excisão de tecido gorduroso ou lipoaspiração conforme esquema adaptado por Cs Nagy et al.^8^ (Figura 1). A implantação de guia subcutâneo [Venous Window Needle Guide^®^ (VWING); Vital Access Corp, Salt Lake City, Utah] também já foi relatada^8-11^ .

**Figura 1:** Opções cirúrgicas para favorecer punções de veias de escoamento de fístulas arteriovenosas profundas em membros superiores.

O presente trabalho adotou o conceito de lipectomia conforme descrito por Bourquelot et al.^12^ , pois é um método atualmente aceitável de superficialização da veia cefálica em FAVs nativas. Ele oferece um procedimento relativamente simples e de baixo custo para a canulação confiável do acesso em pacientes obesos^12^ .

Neste estudo, objetivamos revisar a experiência de um serviço com a lipectomia de acessos autólogos envolvendo a veia cefálica no antebraço e braço de pacientes obesos.

## METODOLOGIA

### Desenho do estudo

Tratou-se de uma pesquisa transversal, retrospectiva e descritiva realizada no serviço de Cirurgia Vascular do Hospital de Clínicas Gaspar Vianna (HCGV). O levantamento do *corpus* de pesquisa foi realizado em banco de dados de confecção consecutiva de FAV para hemodiálise em um hospital público estadual terciário de referência na cidade de Belém, no estado do Pará. Este estudo tem o intuito de delinear o perfil dos pacientes, a segurança e a eficácia da lipectomia como alternativa de superficialização de FAV em pacientes obesos em um centro de referência na Amazônia. A metodologia aplicada seguiu os preceitos contidos no documento *Guidelines on good publication practice*, elaborado pelo Committee on Publication Ethics (COPE).

### Cálculo amostral

O tamanho da amostra foi definido através da fórmula para população finita^13,14^ , com o poder do teste de 0,95, considerando a probabilidade do evento de 0,5, ou 50%, e adotando o nível de significância α = 0,05. No período considerado como parâmetro (60 meses), foram atendidos 328 pacientes, logo, para os 3 meses de coleta, a quantidade de pacientes esperada foi 18. O cálculo apontou um número amostral mínimo de 17 pacientes.

### Aspectos éticos

O projeto foi submetido previamente ao Comitê de Ética em Pesquisa (CEP - HCGV) e seguiu a Resolução n.º 466/2012 do Conselho Nacional de Saúde, preservando sigilo e anonimato. O estudo foi aceito pelo CEP com o parecer substanciado 4.263.413 e o seguinte Certificado de Apresentação de Apreciação Ética: 36268220.8.0000.0016.

### População do estudo

Foram incluídos na pesquisa todos os pacientes renais crônicos dialíticos matriculados na instituição e que aceitaram participar do estudo (com assinatura do termo de consentimento livre e esclarecido) com FAVs nativas radiocefálica ou braquiocefálica disfuncionais por obesidade do membro ou profundidade da veia superior a 6 mm. Por outro lado, foram excluídos da pesquisa aqueles com FAV disfuncionais por outras razões, conforme fluxograma baseado na diretriz STROBE, demonstrado na Figura 2. O estudo foi limitado às fístulas radiocefálicas e braquiocefálicas realizadas no período de janeiro de 2018 a dezembro de 2022.

**Figura 2:** Fluxograma de seleção, exclusão e inclusão de casos selecionados.

### Avaliação dos pacientes

Conforme rotina do serviço, os pacientes foram submetidos inicialmente a uma anamnese e a um exame físico que incluiu inspeção das extremidades superiores para edema, assimetria de pulso, pressão arterial e realização do teste de Allen. A ultrassonografia com Doppler foi realizada rotineiramente em todos os casos antes da criação do acesso.

### Procedimentos cirúrgicos

Em todos os pacientes, o procedimento de confecção do acesso foi realizado em duas etapas. No primeiro momento, uma FAV autóloga foi criada na região do antebraço (radiocefálica) ou em prega cubital (braquiocefálica). No caso de falha precoce do acesso (trombose), uma segunda fístula foi criada alguns centímetros proximalmente, a menos que fossem encontradas lesões ateroscleróticas avançadas ou pequenos diâmetros arteriais. Nesse caso, passamos a usar a artéria braquial na prega cubital. Antes do segundo estágio, os pacientes passaram por avaliação ultrassonográfica, e foram obtidas informações quanto ao diâmetro e à profundidade do vaso (Figura 3).

**Figura 3:** Aspecto ultrassonográfico pré-operatório: imagem transversal incluindo desde a pele até a veia cefálica no braço. Os pontilhados indicam a distância pele-veia acima de 6 mm. As legendas ilustram achados na imagem: FS: fáscia superficial; TCS: tecido celular subcutâneo; VC: veia cefálica; MUSC: músculo.

No segundo estágio, realizado de 4 a 8 semanas após a formação da fístula, um procedimento de lipectomia foi realizado para promover a superficialização da veia e permitir punção segura.Isso foi feito com duas incisões transversais de aproximadamente 5 cm, conforme descritas por Bourquelot et al.^12^ para remoção do coxim gorduroso (Figura 4). Não foi realizada hemostasia preventiva com faixa de Esmarch ou manguito pneumático. No segundo estágio, todos os pacientes receberam protocolo anestésico de bloqueio de plexo e sedação e dose de antibiótico profilático. Por fim, usamos rotineiramente um dreno de sucção contínua a vácuo em sistema fechado (Portovac^®^) de 3,2 mm por 24 a 48 horas e fechamento com fio inabsorvível em pontos separados, completando o procedimento.

**Figura 4:** A visão intraoperatória mostra as incisões para lipectomia em fístula arteriovenosa braquiocefálica de membro superior esquerdo e o coxim adiposo (tecido celular subcutâneo) recém-removido. TCS: tecido celular subcutâneo.

### Seguimento dos pacientes

O exame de acompanhamento de rotina para todos os pacientes com FAV e que passaram por lipectomia incluiu uma breve avaliação por ultrassom do diâmetro da veia, localização e volume do fluxo antes da liberação do acesso. As medidas de profundidade da veia nos mesmos locais foram reavaliadas para cada paciente após a intervenção, e o novo acesso maduro foi mapeado e marcado para a equipe de enfermagem responsável pela canulação. A Figura 5 ilustra um paciente no 28º dia de pós-operatório de lipectomia e já em uso do acesso.

**Figura 5:** Paciente com obesidade grau I no 28º dia de pós-operatório de lipectomia e já em uso do acesso. Os pontilhados indicam incisão de lipectomia já cicatrizada.

### Dados coletados

Foram coletados dados como sexo, idade, tempo em hemodiálise, número de acessos prévios, tipo de fístula, IMC, tempo para liberação de uso do acesso, diâmetro médio e profundidade da veia ao ultrassom, complicações e perviedade.

### Análise estatística

As informações da caracterização amostral foram apuradas e sistematizadas em planilha elaborada no *software* Microsoft Excel^®^. A estatística analítica utilizada para avaliar os resultados das variáveis da amostra ocorreu por meio dos testes G e qui-quadrado de aderência para tabelas univariadas. As estatísticas descritiva e analítica foram realizadas pelo *software* BioEstat^®^ versão 5.4. Para a tomada de decisão, adotou-se o nível de significância α = 0,05, ou 5%, sendo sinalizados com asterisco (*) os valores significantes.

## RESULTADOS

O presente estudo retrospectivo incluiu 22 pacientes obesos (15 homens e 7 mulheres), selecionados entre um grupo de 328 pacientes submetidos à criação de FA autógena entre janeiro de 2018 e dezembro de 2022. A criação de FAV autógena foi bem-sucedida no intraoperatório em todos os casos.

A distribuição do influxo das FAVs funcionantes foi a artéria radial proximal em 4 pacientes (18,2%) e a artéria braquial em 18 (81,8%). Não tivemos síndrome do roubo que justificasse intervenção em nenhum caso. Houve predomínio do sexo masculino, e a média de idade foi de 58,4 anos. A causa prevalente de doença renal terminal foi diabetes melito em 59% e hipertensão em 38% dos pacientes. As comorbidades foram observadas em 95,5%, sendo as principais hipertensão (63,6%), diabetes (54,5%) e cardiopatia (31,8%). Em relação ao tempo em hemodiálise, a média foi de 4 meses, e 88,8% dos pacientes passaram por superficialização no primeiro acesso autógeno. O IMC médio foi de 34 kg/m^2^, sendo que a maioria encontrava-se na classificação nutricional de obesidade grau I. O tempo médio entre a confecção do acesso e a lipectomia foi de 45,1 dias, e o tempo de intervenção (lipectomia) até a liberação para uso nas sessões de hemodiálise oscilou de 21 a 42 dias, com média de 30,9 dias. Uma tentativa prévia malsucedida de acesso distal ocorreu em seis pacientes (22,7%). A lipectomia foi realizada em 14 pacientes (63,6%) sem antes realizar qualquer tentativa de punção, ao passo que 8 pacientes (36,3%) foram submetidos a tentativas malsucedidas de punções e encaminhados para o procedimento (segundo estágio).

As complicações, todas de baixa morbidade, ocorreram em nove pacientes (40,9%), sendo que as principais observadas foram seroma (22,7%) e hematoma (22,7%). Dois pacientes apresentaram necrose superficial de pele e um evoluiu com hemorragia e necessitou de revisão cirúrgica. Não houve infecção ou deiscência de feridas. Três pacientes tiveram atrasos na primeira canulação por conta de hematoma e um, por seroma significativo. As lipectomias foram realizadas em média 45,1 dias após a criação (variação de 27,0 a 90,0). A profundidade média pré-operatória da veia cefálica no membro foi de 7,9 mm (variação de 7,0 a 10,0 mm). Isso foi reduzido para uma profundidade média de 4,7 mm (faixa de 3,0 a 6,0 mm).

O período médio de seguimento dos pacientes foi de 13,2 meses. Houve quatro óbitos durante o acompanhamento, não relacionados ao acesso vascular (dois por infarto agudo do miocárdio, um após acidente vascular encefálico isquêmico e outro por complicações infecciosas na unidade de terapia intensiva), e perda de seguimento de quatro pacientes. Três pacientes passaram por intervenções endovasculares para manutenção do acesso para diálise. Aos 12 meses, a permeabilidade primária após a lipectomia foi de 66,6%, e a permeabilidade secundária foi de 75%. O perfil dos pacientes é demonstrado nas Tabelas 1 e 2, assim como os dados relacionados aos acessos envolvidos no estudo.

**Tabela 1:** Perfil dos pacientes submetidos a lipectomia no período de janeiro de 2018 a dezembro de 2022 no Hospital de Clínicas Gaspar Vianna (HCGV), Belém, Pará.

**Variáveis**	**Frequência**	**% (N = 22)**
Sexo		
Feminino	7	31,8%
Masculino*	15	68,2%
Faixa etária		
< 50	4	18,1%
50 a 59	8	36,4%
60 a 69	10	45,5%
Mínimo/média/máximo	33/58,4/69	
Classificação nutricional		
Sobrepeso	2	9,1%
Obesidade grau I**	13	59,1%
Obesidade grau II	6	27,3%
Obesidade grau III	1	4,5%

* p = 0,0004, teste do qui-quadrado de aderência;

** p = 0,0015, teste G de aderência.

**Tabela 2:** Descrição das variáveis relacionadas aos tipos de acessos, profundidades pré e pós, tempo de intervenção até liberação e seguimento dos casos submetidos a lipectomia no período de janeiro de 2018 a dezembro de 2022 no Hospital de Clínicas Gaspar Vianna (HCGV), Belém, Pará.

**Variáveis**	**Frequência**	**% (N = 22)**	**Valor de p**
Tipos de fístula arteriovenosa (FAV)			0,0028*
Braquiocefálica*	18	81,8%	
Radiocefálica	4	18,2%	
Profundidade pré (mm)			
Mín./média/máx.	7,0/7,9/10,0		< 0,0001**
Profundidade pós (mm)			
Mín./média/máx.	3,0/4,7/6,0		
Tempo FAV até lipectomia (dias)			------
Mín./média/máx.	27,0/45,1/90,0		
Tempo de lipectomia até a punção (dias)			------
Mín./média/máx.	21,0/30,9/42,0		
Seguimento (meses)			------
Mín./média/máx.	2,0/13,2/22,0		

* Teste G de aderência;

** Teste t de Student pareado.

## DISCUSSÃO

A Organização Mundial de Saúde estima que 2,3 bilhões de pessoas estejam acima do peso e que 700 milhões sejam afetadas pela obesidade até 2025^15^. Um relatório recente sugere que, se o aumento nas taxas de sobrepeso e obesidade continuar no nível atual, aproximadamente 85% dos Estados Unidos estarão com sobrepeso ou obesidade até 2030, com uma projeção de 100% até 2048^16^ .

Os dados são alarmantes, pois a estreita relação entre obesidade, hipertensão arterial e diabetes está intimamente ligada com o desenvolvimento de doenças renais, inclusive doença renal crônica (DRC)^5,14,17-19^. No Brasil, homens e mulheres são igualmente afetados, e a obesidade já se aproxima de proporções epidêmicas, com aproximadamente 20% dos indivíduos adultos apresentando IMC igual ou superior a 30 kg/m^2^. A região amazônica não apresenta dados oficiais quanto à prevalência de DRC terminal *versus* obesidade, mas a média de sobrepeso e obesidade é semelhante às médias de demais estados brasileiros^5^ .

Pacientes obesos e diabéticos pertencem a um grupo com risco aumentado de colocação de FAV autóloga^1-4,18,19^. Uma FAV madura, com bom funcionamento e não canulável por obesidade do membro, não oferece nenhum benefício e leva a uma dependência prolongada do cateter e a suas potenciais complicações, com frustração para o paciente e para a equipe de suporte à saúde^6-12,18,19^. Este estudo evidenciou uma profundidade média pré-operatória da veia cefálica de 7,9 mm, que foi reduzida para uma média de 4,7 mm no controle pós-intervenção. Já as complicações ocorreram em nove pacientes (40,9%), todas consideradas de baixa morbidade e que não impactaram no prolongamento de tempo para liberação do acesso.

Assim, pacientes obesos frequentemente necessitam de procedimentos adicionais devido à profundidade da veia, para favorecer uma maturação adequada da FAV^2-4,19^. Isso se soma a maiores taxas de falhas devido a trombose ou disfunção^4,18^. Paradoxalmente, pelas mesmas razões, muitos pacientes obesos têm veias cefálicas consideradas adequadas e que são poupadas de danos repetidos no período pré-dialítico devido às suas localizações inacessíveis^4,11,19,20^. O ultrassom permite uma avaliação precisa, segura e conveniente dessas veias para possibilidade de uso na criação de acessos autógenos^19-21^ .

As diretrizes da National Kidney Disease Outcomes Quality Initiative e Fistula First endossam a chamada “regra dos 6s”, quando a FAV tem diâmetro maior ou igual a 6 mm, profundidade inferior a 6 mm e com fluxo de acesso maior que 600 mL/min. Além disso, um comprimento de zona de canulação funcional de 6 cm também é frequentemente recomendado^7,11^. Desta forma, a recomendação para canulação de acesso confiável sugere uma profundidade máxima da veia de 6 mm^11^ .

A superficialização da veia arterializada, para favorecer a canulação da FAV, pode ser realizada por diferentes técnicas como transposição de túnel, simples elevação da veia ou lipectomia^1,9,11-19,22^. A implantação de um guia de agulha subcutânea (VWING) também foi relatada recentemente por Hill et al.^23^ .

O conceito de lipectomia mini-invasiva foi introduzido por Zeindler et al.^24^. Eles apresentam a lipoaspiração realizada endoscopicamente como opção técnica para reduzir a camada adiposa sobre a veia a ser puncionada no membro. Mesmo sendo uma série de casos, o mesmo grupo de autores relata resultados que podem chamar atenção, pois retratam a realidade da disfunção do acesso em 12 pacientes obesos com profundidade venosa média de 10,1 mm.

A técnica, embora inicialmente associada à necrose da ferida e a grandes hematomas, na atualidade tem sido apresentada como alternativa a correção da disfunção do acesso em pacientes obesos. Ela propõe minimizar o trauma tecidual e favorecer resultados funcionais e estéticos^25^. Além disso, traz vantagens em relação às superficializações convencionais: menores incisões e potencial para reduzir as complicações da ferida, mas, para o procedimento, deve ser consideradas questões como custos, necessidade de habilidade em cirurgia endoscópica e curva de aprendizado, pois não está no arsenal de muitos cirurgiões vasculares no Brasil^4^ .

A tendência para intervenções minimamente invasivas é crescente em diversas especialidades. No âmbito da cirurgia vascular, caminha pelos procedimentos endoluminais, embora haja um potencial sinérgico das técnicas. Assim, no futuro, especula-se que FAVs percutâneas com superficialização endoscópica podem se tornar uma realidade para pacientes obesos com DRT^1^ .

Já na técnica proposta por Bourquelot et al.^12^ em 2009 a veia cefálica é deixada intacta em sua posição nativa, o que reduz risco de torção ou angulação de uma veia reposicionada. Foram relatados excelentes resultados de perviedade primária e secundária em 1 ano, de 71 e 98%, respectivamente^12^. Finalmente, o uso de anestesia locorregional na maioria dos pacientes obesos com DRC terminal é uma grande vantagem. Foi o método de escolha adotado em 100% dos procedimentos realizados no serviço.

Embora as diretrizes atuais da European Society for Vascular Surgery (ESVS) não definam estritamente um nível de IMC que seja uma contraindicação para a criação de fístula, um procedimento cirúrgico primário ou secundário para favorecer a maturação vem sendo defendido pela ESVS, pois a perviedade da FAV em longo prazo é pior na população obesa do que na população não obesa e tem uma taxa de falha secundária mais alta^1,4,7^ .

Tordoir et al.^6^ conduziram uma revisão sistemática da literatura com diferentes técnicas para favorecer canulações de FAV autógenas para hemodiálise. Eles selecionaram 17 estudos relacionados ao tratamento de complicações de canulação e verificaram que a lipectomia resultou em uma taxa de sucesso inicial de 100% com perviedade primária e secundária de 71 e 98%, respectivamente, após 1 ano de acompanhamento^6^. No presente estudo, a perviedade primária foi definida como o tempo (em meses) com perviedade ininterrupta e sem necessidade de intervenção^20-22^. A perviedade primária assistida foi o tempo de perviedade ininterrupta da construção original da FAV, na qual qualquer procedimento intervencionista adicional foi necessário. Já a perviedade cumulativa (secundária) foi o período desde a construção original da FAV, na qual a interrupção da FAV foi induzida por trombose, com ou sem recuperação da FAV, até o abandono do acesso^12,20-22^. As taxas de perviedade primária, primária assistida e secundária no período de estudo foram, respectivamente, de 66,6, 70,2 e 75%.

A obesidade na população em hemodiálise é aproximadamente o dobro da população em geral, e a lipectomia é um recurso técnico adotado para favorecer a canulação nesse grupo de pacientes^6,9-12,17,20,21^. No entanto, alguns pacientes na faixa de sobrepeso, com IMC de 28 kg/m^2^, também passaram pelo procedimento em virtude da grande distância entre a pele e a veia cefálica. Em nossa amostra, isso ocorreu em 9,1% dos casos e em acesso proximais à prega do cotovelo, local em que a veia cefálica corre relativamente profunda ao se aproximar do cotovelo ou quando a dilatação esperada da veia parece ser dificultada por uma fáscia superficial espessa aprisionando a veia.

Não há consenso na literatura sobre a lipectomia primária ou em segundo estágio. Elbarbary^10^ estudaram a viabilidade e segurança da lipectomia realizada na mesma sessão com a criação de fístula de hemodiálise em pacientes com veias cefálicas profundas em comparação com a lipectomia secundária. O sucesso técnico imediato foi alcançado em todos os casos em ambos os grupos. O sucesso clínico não foi significativamente diferente entre os grupos^10^. A taxa de permeabilidade primária cumulativa foi de 88% em 1 ano, consistente com resultados de lipectomias realizadas em segundo estágio. Enquanto isso, as complicações pós-operatórias foram em sua maioria leves, sem diferença significativa entre lipectomia primária e secundária^21-24^ .

A maioria dos autores relatou melhora do diâmetro, da profundidade e boa perviedade das veias após procedimentos de superficialização secundária, mas Elbarbary^10^ questionaram o tempo desde a criação da fístula até a canulação bem-sucedida, o que pode impactar em maior tempo de uso de cateter venoso central. No presente trabalho, esse tempo médio foi de 76 dias, em que 100% das lipectomias foram realizadas em segundo estágio. Isso é especialmente importante em países onde o encaminhamento para cirurgia ocorre imediatamente antes ou mesmo após o início da hemodiálise, o que não reflete a realidade da região amazônica, onde os casos operados estavam em média há 4 meses em hemodiálise.

A lipectomia primária tem a vantagem adicional de operar em uma área virgem, evitando a dissecção sobre uma veia aderente com hematoma subjacente ou fibrose de tentativas anteriores de canulação, observadas em alguns casos de lipectomia secundária^9^. Isso ocorreu em oito casos (36,3%), podendo acarretar complicações pós-operatórias pelas dificuldades impostas durante a dissecção.

Por todas essas razões, a indicação de lipectomia não é um consenso na literatura, mas parece ser preferencialmente uma intervenção de segundo estágio, realizada somente se o acompanhamento clínico e de imagem evidenciar a necessidade para superficialização. Além disso, as experiências com superficializações de FAV parecem ter uma execução mais simples quando o diâmetro da veia já aumentou e a parede da veia se tornou mais espessa pelo processo de arterialização.

Os resultados do presente artigo devem ser interpretados no contexto de suas limitações. O estudo é de natureza retrospectiva, com período de acompanhamento relativamente curto, sem grupo de comparação e com risco de viés de seleção. Por esse motivo, a recomendação geral, de que todos os pacientes obesos com FAV profundamente localizadas devem ser submetidos a lipectomia, não pode ser afirmada.

Este estudo traz a experiência de um serviço de referência na Amazônia e resume as evidências disponíveis sobre métodos de superficialização com lipectomia em estudos que tratam de pacientes obesos. Ele traz, adicionalmente aos especialistas no cenário nacional, a oportunidade de ampliar o leque de opções de procedimentos em acessos disfuncionais por obesidade do membro e reforça a necessidade dos cirurgiões em considerar esses aspectos para planejar e alinhar expectativas junto aos pacientes.

## CONCLUSÃO

A obesidade não deve ser um fator limitante para a criação de uma FAV nativa, pois a lipectomia é uma alternativa promissora, relativamente simples e importante no arsenal cirúrgico de superficialização, permitindo a funcionalidade de FAVs nativas e profundas nesta população. Na experiência do serviço, o tempo médio entre a criação do acesso e a lipectomia foi de 45 dias e de mais 30 dias desde a intervenção até a liberação para canulação, cumprindo o propósito maior de retirar um coxim gorduroso de 7,9 mm em média para 4,7 mm (inferior a 6 mm) entre a pele e veia.
